# Distributed rewiring model for complex networking: The effect of local rewiring rules on final structural properties

**DOI:** 10.1371/journal.pone.0187538

**Published:** 2017-11-06

**Authors:** Magali Alexander López Chavira, Ricardo Marcelín-Jiménez

**Affiliations:** Department of Electrical Engineering, Universidad Autónoma Metropolitana, Iztapalapa, Mexico City, Mexico; Universidad Rey Juan Carlos, SPAIN

## Abstract

The study of complex networks has become an important subject over the last decades. It has been shown that these structures have special features, such as their diameter, or their average path length, which in turn are the explanation of some functional properties in a system such as its fault tolerance, its fragility before attacks, or the ability to support routing procedures. In the present work, we study some of the forces that help a network to evolve to the point where structural properties are settled. Although our work is mainly focused on the possibility of applying our ideas to Information and Communication Technologies systems, we consider that our results may contribute to understanding different scenarios where complex networks have become an important modeling tool. Using a discrete event simulator, we get each node to discover the shortcuts that may connect it with regions away from its local environment. Based on this partial knowledge, each node can rewire some of its links, which allows modifying the topology of the entire underlying graph to achieve new structural properties. We proposed a distributed rewiring model that creates networks with features similar to those found in complex networks. Although each node acts in a distributed way and seeking to reduce only the trajectories of its packets, we observed a decrease of diameter and an increase in clustering coefficient in the global structure compared to the initial graph. Furthermore, we can find different final structures depending on slight changes in the local rewiring rules.

## Introduction

In this work we study the structural properties of complex networks, highlighting the importance of the local actions or decisions taken by the individuals of the system, in the shaping of the resulting structure. Some works in complex networks have analyzed the underlying structures in existing systems and their influence on the dynamics on the network, for example, the network formed by neurons of the brain and the connections among them [1]. In contrast, our study focuses on the dynamics of rewiring and the resulting structure that emerges. This approach matches with different studies that have been proposed to produce networks with specific characteristics, such as the short distances between nodes, these models try to explain how the links in real networks are formed.

### Related literature

Since the end of the last century, a new set of very effective models and tools have emerged to address problems from different domains, such as physics, biology or economics [2]. The objects studied by this new body of knowledge are known as complex systems. In [3] Mitchell proposes that a complex system is formed by a massive number of entities or components that interact without the mediation of central control, only following a simple set of operational rules by which, however, they achieve a very elaborate collective behavior. This emergent behavior, as it is called, is the result of information processing and adaptation, based on learning or evolution. We say, on the other hand, that the interactions between its components occur at a microscopic level. However, at a macroscopic level, they all show a behavior or emerging structure capable of adapting to changes over time. It is also known that the construction of the Internet [4] and the World Wide Web [5], for instance, obey these premises and exhibits the structure of complex networks. We know that this structure gives them special features, such as their diameter and fault tolerance, but it is also the cause of their fragility under attacks.

In information and communication technologies (ICT) there are systems in which the presence of graphs is immediately recognized, for example, the topology of connections in a telecommunications network or the topology of interactions between the processes of a parallel system. Complex networks have become an important tool to study and improve these systems, for example regarding efficiency, allowing the creation of a base (structure or topology) that allows maximization of the capabilities of each component.

Complex networks may contain thousands or millions of nodes, for example, the network that models global society. The connections between people arise from individual decisions that can be taken through a dynamic process that, over time, generate benefits for the individuals and the society of which they belong to. In 1967, Milgram found that the diameter of social network can be very small, compared to the order of the underlying graph. He called this feature the small-world phenomenon [6]. This study revealed some remarkable facts about the social structure: not only there exist short paths between any two persons (i.e., it is a small world), but also people operating only with local information are very adept to find them (i.e., this small world is easily navigated). It is also worth mentioning the study of Granovetter, where it is underlined the convenience of individuals on having long distance acquaintances [7]. Indeed, the experiment of Milgram reveals the first forwarding step in the path of the messages is the longest, while the remaining are refinements in the target search. On the other hand, we know that a complex network may evolve over time and from this perspective can be understood as a dynamic process.

Different models have been proposed to build networks with specific characteristics, such as the short distances between nodes described in the experiments mentioned above. In 1959 Erdös and Rényi introduced one of the first models to generate random networks [8]. This graph model is called Erdös-Rényi model. The set of networks formed under this model are known as ER random graphs identified as *ER*(*n*,*p*), which describes the set of graphs of *n* vertices in which each possible pair of vertices is connected with probability *p*. The degrees of the vertices of a random network follows a binomial distribution that tends towards a Poisson distribution for large values of *n*, the average path length is $\frac{log(n)}{log(np)}$, and its clustering coefficient is defined by the connection probability *p* [9]. The best-known models for complex network generation are the small-world and the scale-free models. In 1998, Watts and Strogatz developed a model for network creation whose key features are: first, to have a small diameter on the order of *O*(*log*(*n*)) where *n* is the total number of nodes in the network. Secondly, a high clustering coefficient given by $\frac{3(k-2)}{4(k-1)}$ where *k* is the number of edges that have each node, and finally an exponential degree distribution [10]. The graphs created with this model are called Watts-Strogatz or small-world graphs (SW). In this model there exist an initial graph of fixed order and symmetrical structure, then each of its vertices is randomly rewired under a given probability.

There are networks in the real world that have characteristics that cannot be fully represented by the Watts-Strogatz model. In 1999 Barabási and Albert developed a study in which it was observed that the distribution of degrees of many networks follows a power-law distribution [11]. Networks that have this type of distribution are also called scale-free networks which means that we observe the same relative degree distribution at any scale. The graphs generated by the Barabási-Albert model (BA) show an important property that is the arising of a very small subset of nodes with a very high number of connections, called *hubs*. The Internet has a scale-free structure. Barabási and Albert provide us with the preferential attachment rule, in which the connection is understood as a dynamic process that occurs when new nodes are added to the existing graph. The probability of an existing node to receive new connection proposals depends on its current degree. The distribution of degrees of BA graphs is given by *P*[*k*] ∝ *k*^−3^. The average path length is given by $\frac{log(n)}{log(log(n))}$ and the clustering coefficient is $\frac{{\left(log\left(n\right)\right)}^{2}}{n}$ [9].

We can recognize important properties for its direct impact on the functions of ICT systems, which are affected by the underlying structure of a given complex network: the navigability of a certain network or its robustness under a degradation process are only two important examples.

In 2000 Kleinberg reviewed the Milgram experiment and concluded that, in addition to the existence of small paths in the underlying graph, the individuals were able to find them from local information, without having a global knowledge of the structure that connects them. He found that the network structure helps to find the existing short paths (making it navigable), but only a subset of the small world networks have this property. Taking this into account, he proposes a model to construct navigable networks [12]. A distributed search executed on this type of structure is bounded by the (*log* (*n*))^2^, where *n* is the order of the underlying graph. This feature is of great importance in information technology applications. If we want to support content search in a Peer to Peer network (P2P), for instance, it would be of great help to know that the search is performed on top of a navigable network.

Another important property of an ICT system is the ability to maintain the connectivity of a communications network, while it undergoes a degradation processes such as intentional attacks or random failures of its nodes. A fault occurs when a random element in the network stops its operation or is removed. An attack happens when an element is chosen to be eliminated according to its characteristics, to destroy or cause the biggest damage on the overall structure. Albert, Jeong and Barabási, studied the behavior of different types of networks, while their structures undergo a degradation process [13]. They found that networks with power-law degree distribution have a high fault-tolerance. In contrast, these structures are very vulnerable to attacks since there exist a minority of highly connected nodes, called hubs. Also, Albert et al. found that networks with an exponential degree distribution have a higher attack tolerance than those with a power-law degree distribution, but their fault tolerance is not as good. It is also worth mentioning the works of Cohen et al., who analyzed how a network responds under constant failures [14]. These authors studied the Internet structure that follows a power-law distribution. They showed that Internet is highly resistant to failures but under intentional attacks, they found that the network is very sensitive to this type of degradation process [15]. It only takes a very small number of hubs to get the network dismantled into isolated components. The moral is that these hubs are the key to achieve very short paths since they act like bridges connecting distant regions, otherwise isolated. Therefore, the key to a network with short path lengths seems to be, at the same time, the source of the major network vulnerability.

In the work of Leduc and Thurner [16] we observe how a rewiring process is deployed to obtain a beneficial feature on the structure of the network. They studied a financial network formed by banks and transactions between them; the main goal is to generate a resilience network against cascading faults that may collapse the overall system based on the taxes used in financial transactions. In this work, we observe how the information contained in the network can be analyzed to rewire the structure to obtain a benefit in its characteristics. In this case, decisions are taken individually, but based on measures which have been assessed centrally.

The formation of a complex network can be understood as the result of a process in which each element takes its decisions based on a partial knowledge that, however, has a global impact. This approach is interesting since it can be observed that many of the real-life networks are formed through the interaction between their elements following these types of rules. In the work of Ch’ng [17], for instance, we can observe how the interactions among the users of a social network app produce an emerging structure. Ch’ng showed that events on Twitter, whether a special topic or news, along with the activity of the comments made by the community, create a small world network. On this context, the small world phenomenon may help to understand the development of social movements.

We have found many efforts to introduce complex network structures into ICT systems, for example, in P2P networks, as we have already mentioned [18, 19]. In all cases, the underlying structure is required to fit the purpose of the system. In Marza et al., for instance, the authors propose a video streaming system, supported by a hybrid structure combining features from small-world and scale-free networks. Also, they propose a node aggregation rule in which each node takes into account its geographical position [20]. The model they propose generates a more robust and fault-tolerant structure. The authors compared their solution with small-world structures, scale-free networks and structured networks, showing that their model has the shortest path lengths, and the highest clustering coefficients.

Also, it is worth mentioning the work of Manku et al., where authors introduce the Symphony protocol, which builds a Watts-Strogatz network topology. Symphony was developed to improve the scalability and search complexity in the resulting network [21]. A dynamic P2P network is managed using Distributed Hash Tables, which contain updating status information. When a change occurs in the network structure, either by adding or deleting a device or object, the hash tables containing the indexes of the stored data must be updated. Such operations generate substantial costs that limit network scalability. The goal of Symphony is to speed up the updating task inspired by the ideas of Kleinberg. The resulting network meets the criteria of scalability, stability, performance, flexibility and simplicity. The authors showed using simulations that, with a constant number of long-range links equal to *K* = *O*(1), it is possible to redirect hash searches with an average latency of $O(\frac{1}{k(log_{2}\left(n\right))})$ jumps. As a consequence, they also obtained a network where each node requires a rather small set of links.

Ripeanu et al., explored the Gnutella network [22] using a tracker, and found that its structure is close to a scale-free model, which has an impact on its performance and reliability. In the downside, they found that the generated traffic limits the scalability due to the existence of hub nodes that concentrate a very high traffic load. Also, scale-free networks have been proposed to support Massive Multiplayer Online Games over P2P networks. Currently, these games run on centralized systems. Using P2P networks could improve this service and lower the cost to the final user. In the work of Ferretti and D’Angelo, for instance, we can see that the diameter of the scale-free network allows the updates of the game to reach all players within a few leaps [23]. Also, the topology becomes highly fault tolerant but, as we now know, very vulnerable to intentional attacks that may compromise the service.

The work of Qi et al. presents a different viewpoint from the studies mentioned above [24]. In this case, a scale-free network is dynamically built, using a modified Barabási-Albert model. In the new approach, besides newcomers, new edges are created between nodes previously deployed. Both models, BA and the modified BA, were compared to a real electronic commerce network in which it is observed how the clients build links among them. The result of this experiment indicates that as the scale of the network increases, the number of vertices with a high degree remains unchanged, while the clustering coefficient and the average path length decrease to reach constant values. Also, it was observed that the resulting structure has a better resemblance to real-world networks. The analysis performed in [24] has provided us with a lot of ideas that we incorporate in our toolkit.

In the work of Guclu and Yuksel [19], we see an example of how to settle local rules to induce a scale-free network structure over a P2P system. They introduced cut-off rules in the degree of each node, that is, limiting the maximum number of links that a node accepts. These cut-off points avoid the formation of hubs with a very high degree which provides scalability to the P2P network. Two mechanisms are proposed to build scale-free networks based on partial information only: HAPA (Hop and Attempt Preferential Attachment) and DAPA (Discover and Attempt Preferential Attachment). Through simulations, they studied the efficiency of different search algorithms on the topologies generated by the proposed mechanisms. They found that the degree distribution exponent, decreases as the cutoff point used in the nodes becomes smaller. Also, they showed that the topologies produced by the proposed mechanisms allow greater efficiency under the search algorithms they tested.

In the case of Colman and Rodgers [25], we see a different proposal in which, the model is based on network rewiring, they have directed networks in which the rewiring can be local or global depending on how is selected the new node in the rewiring phase. The probability of each node being chosen will change as the model advances, and the connectivity of each node in the network is modified. Their main result is the proposed model that generates networks with exponential degree distribution with only a small number of hubs; the rewiring rules avoid the formation of a power-law distribution. Also in this paper, the authors study the network connectivity with different local rewiring rules.

It is important to analyze the tolerance to failures or attacks as an emergent property of these models. In the work of Iyer et al. [26], they study the works of Barabási and Cohen, investigating the effect on the structure of the network while being submitted to different degradation processes, considering the degree and the centrality, among other features. They present a study of the impact of various attack methods on a wide variety of networks. They found that, under simultaneous attacks and for most of the networking models, the most vulnerable vertices are those with the highest degree. In contrast, under sequential attacks and any of the networking models, removing vertices in reverse order of betweenness, turns out to be the most efficient procedure to degrade the appointed structure.

### Aim of the work

We observe that building a complex network may be understood as the result of a dynamic process that shapes its topology over time. We have reviewed several experiments that propose to model this process as a graph in which each node applies a set of rewiring rules. In the present work, we study some of the forces that help a network to evolve to the point where structural properties are settled. Using a discrete event simulator, we get each node to discover the shortcuts that may connect it with regions away from its local environment. Based on this partial knowledge, each node can rewire some of its links, which allows modifying the topology of the entire underlying graph to achieve new structural properties. Our objective is to recognize those local conditions that allow nodes to make decisions from which interesting global properties emerge, such as those that can be found in complex networks. Although our project is mainly focused on the possibility of applying our ideas to ICT systems, we consider that our results may contribute to understanding different scenarios where complex networks have become an important modeling tool. In this sense, we are interested in structures with fast delivery times, this is reflected in small diameters. Other notable features are the formation of hubs or creation of clusters that can impact the fragility or resilience of the corresponding systems.

The rest of this paper includes the following sections. In Section 2, we describe the proposed model of network formation, we describe the working assumptions and the experimentation tool, finally we explain the experiments set. In section 3, we analyze the results of the experiments campaign. Finally, in the section 4 we discuss our results.

## Methods

We have found at least 3 complementary approaches to address the study of network creation: i) physics models, ii) economic models, and iii) agent-based models. In the first case, differential equations model the process and they are intended to find the degree distribution of the nodes after the process has reached a steady state [27]. In the second, game theory models are used to describe how some pairs of nodes decide to connect each other, obeying a set of strategies that seek to optimize their benefit of each of the parties [28, 29]. Finally, in the agent models [30, 31], it is considered that in each node resides an active entity or agent, who decides to establish links with others of its type, to reach a goal. As Axelrod tells us in his work, although the assumptions of the models may be simple, the consequences may not be obvious [30]. It is important to note that so far, none of the approaches are considered preferable over the others. We insist that they are considered complementary ways of studying the same problem. On the other hand, it is also interesting to highlight how the models assume that the decisions are autonomous and are based on the local information available to the nodes, that is, none of the nodes have a global vision.

We considered that there is an opportunity to study massive scale networks, using simulation tools. As mentioned in the previous section, a complex network is the result of a process that shapes the structure over time. We developed a simulation platform in which, departing from a rather regular graph, we observe the way nodes, regarded as agents, try to optimize the path lengths of their messages. Meanwhile, the global structure evolves and turns into a complex network. The design of our simulation platform assumes the dynamic model described below. In this work, we focused on the importance of the local rules of the agents belonging to the simulated system.

### Model and working assumptions

The proposed model is inspired by the ways in which people settle bonds. A person is initially linked to his immediate community, such as his family, neighbors and co-workers. However, as time goes on, he uses these initial links to meet people beyond his first acquaintances and creates long distance ties. The model considers the processes of sending messages that occur in the network so that this exchanging process allows each node to acquire partial information about the underlying structure. Each node uses its neighbors, which make up their immediate community, to find those nodes in the network that are potentially useful for establishing long-range links or shortcuts.

Our experiment evolves through *cycles* or rounds. Cycles are required to enforce some form of synchronization. In turn, synchronization is required to maintain an updated knowledge of the underlying graph. Each cycle is divided into two phases: i) packets exchange and ii) links rewiring. Cycles are repeated as many times as necessary to reach a convergence criterion, which may be based on the differential number of rewiring links between two consecutive cycles. That is, if the number of rewired links in a cycle is close to zero, it means that the network structure is no longer having important changes.

**Fig 1:**
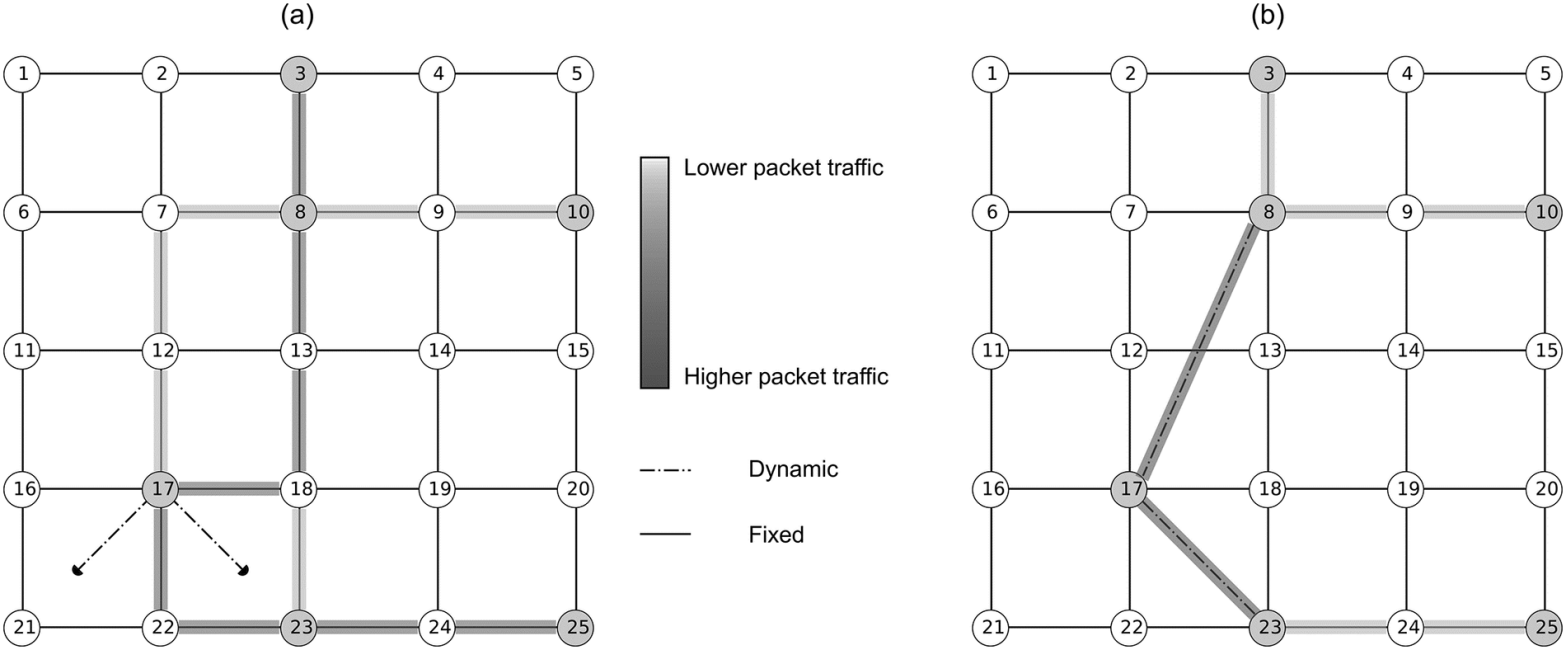
Main idea of edge rewiring. (a) During the exploration phase, each node sends packets to random destinations. In our example, node 17 issues five packets with nodes 3, 8, 10, 23, and 25 as the final targets. (b) When acknowledgments return to the source, the node realizes that its packets might follow shorter paths if it builds a direct connection to nodes 8 and 23 that appear as shortcuts to most of the places that it has recently explored.

Each node has an initial set of links that shares with its corresponding neighbors. This links can be classified in 2 subsets: *fixed* and *dynamic*. During the packet exchange phase, each node uses all of its incident edges to discover a distant node offering a potential decrease in the path lengths of future packets (Fig 1a). During the rewiring phase, each node rewires its less useful outgoing link(s), i. e. that neighbor that routed fewer packets than the rest, to the distant node(s) that offers the best chances of reduce the path lengths of future packets (Fig 1b). Below we describe in detail each of the stages that build the proposed model, the tools used for the construction of the simulator and some of the experiments that show the power of this tool.

On the initial deployment, the whole set of nodes take part of a 2D square grid. Each node has a Von Neumann neighborhood composed of the top, bottom, left and right neighbors, except for the nodes lying on the borders and corners of the grid, which have 3 or 2 neighbors, respectively. This initial structure is only tied by its fixed links. Meanwhile, in this very beginning, the outgoing dynamic edges are initially untied. We proposed a grid of two dimensions because in this topology is easy to know the position of each node. However, it is important to note that any initial topology works, provided that there is a routing mechanism that guarantees the delivery of each issued packet.

Every node takes decisions based on the available knowledge it has about its local conditions. This knowledge is built in 3 different ways: 1) it is wired according to its current links, 2) it is recorded containing its permanent position within a set of global coordinates, 3) it can also be recorded, and updated, in a local log where the node is able to track the whereabouts of the packets that it has sent or received (a node is able to work in 3 different roles: as a source, as an intermediate forwarding node, and as a target or destination).

During the first phase of a cycle, each node creates a set of packets, called *tracers*, with random destinations. Based on its local information, a node in charge (either a source or an intermediate node) forwards a packet to the next step on the route to its final node. In our implementation, the packets are sent one after the other, i.e. when a node receives an acknowledgement that indicates that its tracer has reached its final destination, it will launch the following tracer. However, all packets could be sent simultaneously in our implementation without affecting the results of the model. Our working assumptions imply that each node is aware of its embedding within a metric space. Therefore, routing may use this property. Each node forwards a tracer packet based on its local implementation of the compass routing algorithm [32]. To choose the next step of a packet, the routing is performed as follows: we draw a main straight line between the current node in charge and the final destination. Also, we draw a secondary line between the node in charge and each of its immediate neighbors. Each secondary line crosses the main line with a different angle. The neighbor corresponding to the smallest angle will be designated as the next node in the route of the tracer. Before leaving, the tracer annotates the id of the current node in a list of visited nodes. When the tracer reaches its final destination, the receiver issues an acknowledgement that travels back to the source node, based on the list that the tracer built. In turn, the source takes notes about the places that its initial packet has visited. Notice that each source node can rank the nodes that have been visited by its tracers, from the most visited to the least. Also, each node can rank its dynamic links depending on their utilization. Those that have been frequently used can be classified as valuable links. On the other hand, those that have been seldom used can be classified as worthless links.

While Compass Routing gives good results, it may produce cycles during routing. We use the list of visited nodes to avoid this drawback. If a path does not lead to the final target, we return to the previous decision point and choose another route. Nodes that cause this backtracking decision are recorded to be avoided. If a packet cannot reach its final destination, it will eventually return to its origin. In this condition, the source issues a new tracer. Within the simulator, it is possible to modify the routing algorithm easily.

During the second phase of a cycle, each node reviews how many times its links have been used to route a packet, and the ranking of how many times the nodes which have been visited by its tracers were used. It is important to mention that the most visited nodes do not necessarily represent the final targets of the previous phase, but the potential shortcuts to reach distant regions. Therefore, based on its statistics, each node sends a connection requests to its candidate node. If this node accepts, the home node sends a message to its most useless link to disconnect from it and updates its list of neighbors. Next, the node estimates the benefit it has achieved with this new settlement. This measure can be evaluated in terms of the number of rewired links, the expected reduction on its average path length, the expected improvement on the utilization of its temporary links, or any measure reflecting a change on the local knowledge.

**Fig 2:**
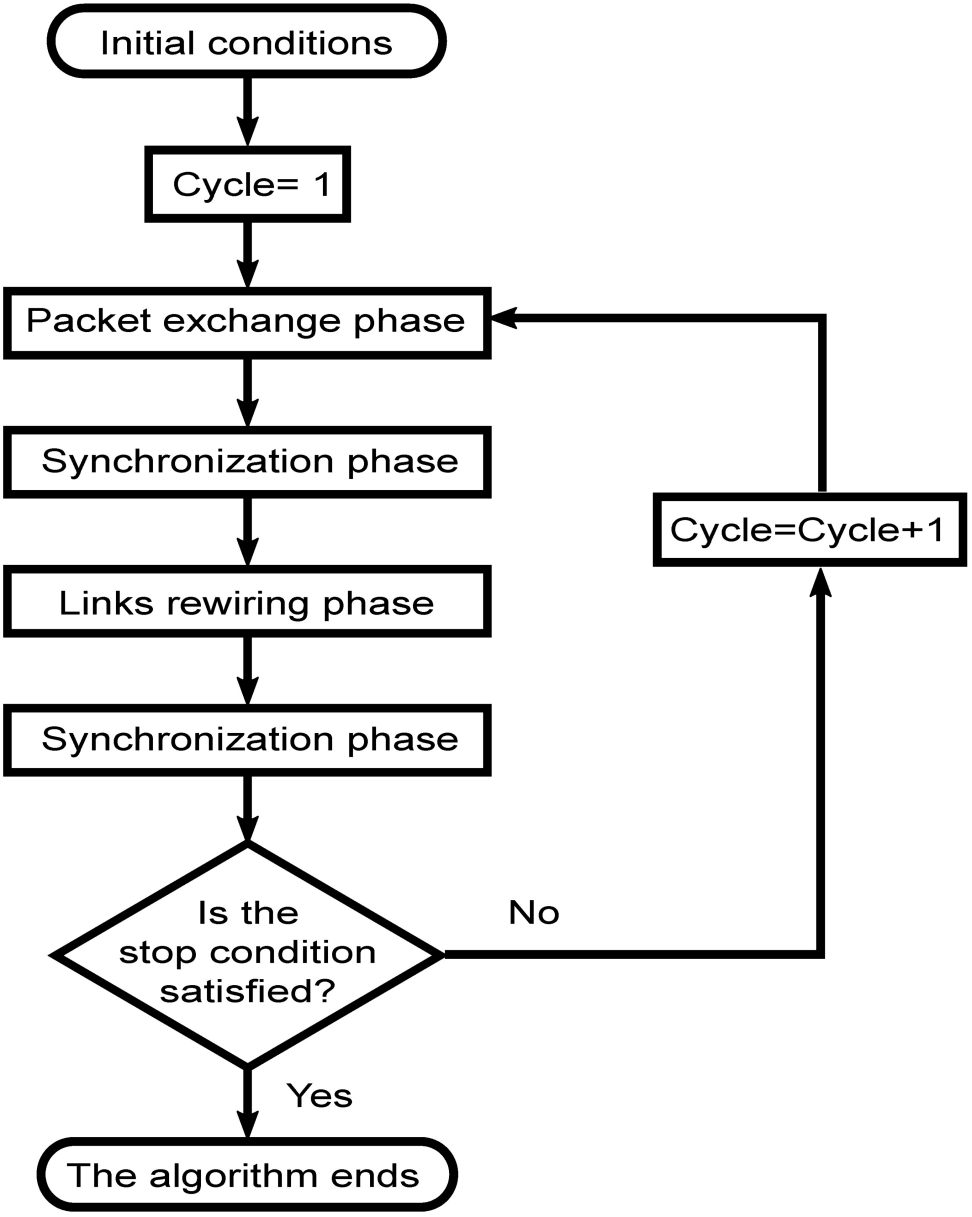
Diagram of the proposed model.

Cycles are repeated as many times as necessary to reach a convergence criterion, which can be based on the differential benefit or the improvement between two consecutive cycles. When this improvement is below a fixed parameter, the coordinator decides to stop the overall work. We can see the interactions between the phases in Fig 2.

### Tools and construction of the experimentation platform

As we mentioned earlier, the use of simulations is a key instrument for scientific research [31]. From this point of view, simulation can be regarded as a valuable tool in experiments where an intervention on the real system is not possible. In the development of our experimental platform we found several options close to the type of tool that we were looking for. One of the most used simulators is NetLogo [33], which allows the programming of agent-based models, including the definition of the rules to be followed by the active entities, within a simple environment that allows visualizing the entire system and supporting the monitoring of agent interactions. Although being a very useful tool, its use was ruled out because each element of the simulation should be programmed as an agent, including links and packets. Considering the large number of vertices to be modeled and particularly the number of exchanged packets, the preliminary experiments deployed with this tool, were too long.

Within the tools for complex networks simulation, we also found GNS3 [34], Pajek [35] and Mason [36]. All of them support the multi-agent paradigm based on discrete event simulators. Also, they separate the visualization from the simulated system, so they became a better option to code the proposed model and to handle large amounts of vertices, however these tools allow us to work solely with the structures and dynamics of the network. For our experiments, we need to model processes that run on the structure and provide nodes with a certain sense of awareness about the information they can get from these processes, to take advantage of the global structure, i. e. a node should be able to react according to the dynamics on the network. Let us recall that in the present work the emerging structure is generated in a similar way to real-world networks, that is, its creation is a consequence of the decisions of independent agents.

In order to build the intended experiments, we realized the necessity of a tailor made tool offering two main features: i) the possibility of coding and deploying distributed algorithms over different underlying graphs, ii) the possibility of recovering structural information after a rewiring phase is finished. Therefore, we built our own tool based on a simulator previously developed in our team by Marcelín-Jiménez [37], supporting these initial requirements. Besides, this tool is coded in Python 2.7, which offers a wide variety of helpful libraries including graph theory analysis. Among the most salient libraries of this kind, we incorporate NetworkX 1.9.1 which provides functions to measure degree distribution, clustering coefficient, diameter, average path length, and connectivity.

### Experiments

We proposed a main simulation scenario. We start with a two-dimensional grid, where each node of the network has two types of links: fixed and dynamic. The former make up the initial Von Neumann neighborhood. The latter can be stretched out up to a limit length, so that each node is able to rewire these links to the nodes within a bounded region only. However, under certain experiments, this limit length allows to reach any node of the grid.

The initial node’s community is formed by the links of the two-dimensional grid and this initial working edges are considered fixed, so the Von Neumann neighborhood of each node will remain throughout the simulation. On the other hand, each node has a set of rewirable or dynamic links that, at the beginning are only connected to the owner node, and can be understood as loose ends.

During the first rewiring phase, if a node has an unallocated link, it will connect it to the vertex that it found to be the most useful within the scope of its stretching links. In the following cycles when all its dynamical links are connected, the node will follow the normal rewiring, moving these links only when it finds a promising candidate.

For the rest of our work we will define two families of experiments based on the following rewiring rules.

**Optimistic condition**: A node performs a rewiring action upon disconnecting from its less-used neighbor and connecting to the most visited node, found during the packet exchange phase.

**Conservative condition**: A node performs a rewiring action upon disconnecting from its less-used neighbor and connecting to the most visited node, found during the packet exchange phase, provided that this candidate offers a higher utility than any of its current neighbors.

In summary, the Optimistic condition has more opportunities to rewire links to places farther away from its original neighborhood than the Conservative condition, which is more restrictive in choosing the nodes to rewire its links. The reason for analyzing these two conditions is that we are interested on the way an agent faces risky or long term decisions in a complex system. From our view, an optimistic node is not a gambler, but it dares to invest on middle or long term possibilities. Meanwhile, a conservative node only considers an immediate reward. We want to observe the impact of this slight constraint on the final structures.

For each of the above conditions, we considered a complementary parameter that defines each of the experiments under study: the maximum length that a dynamic link is able to stretch out. Given D, the distance that separates the right lower node from the left upper node, we decided to study the following stretch lengths: *D*, *D*/2, *D*/4, *D*/8 and *D*/16. The experiments were performed starting with an initial grid of 2500 nodes. Each node sends 20 tracer packets for each cycle and has 2 dynamic links. We perform several experiments, for each rewiring rule, and we found out that the number of exchanged packets on each cycle only influences the time to reach the convergence criteria. Nevertheless, the final graph properties remain the same. Therefore, we conclude that it is the rewiring rule the *force* that shapes the graph. These results offer us the leeway to decide the number of packets exchanged on each cycle as a trade-off between speed and network congestion. At the end of each cycle, we measured the average clustering coefficient (ACC), diameter, average path length (APL) and the number of rewired links, in addition, the distribution of degrees of the final network was obtained. As we mentioned earlier, these measures were assessed using NetworkX. We defined a stopping condition based on the number of rewired links in a cycle. When this number reaches a minimum, we consider that this condition has been reached. According to a preliminary study, we found that after 30 cycles, the rule is fulfilled in all cases that we considered. The code that supports this work is available in: https://doi.org/10.5281/zenodo.996625. And the data is available in: https://doi.org/10.5281/zenodo.996601.

## Results

We separate our experiments in two main sets depending on the rewiring condition. Likewise, within each main set, simulations were performed for each of the proposed stretching lengths in order to observe the effect of this local constraint on the overall characteristics of the final graph. We recognize some general observations on the results of the proposed model simulations. During the rewiring process, we observe in all experiments an increase in the clustering coefficient and a decrease in the diameter and average path length from the initial graph. In addition, we observe that these characteristics change as the stretching of the dynamic link is limited. However, we observed slight differences when we altered some specific rules within the rewiring model.

In Table 1 we observe the first set of experiments, using the Optimistic condition, this table shows the average clustering coefficient, diameter and average path length of the final graphs. In the first row we see the characteristics of the initial two-dimensional graph, in the following rows we have the mean values achieved in the final graphs, each row corresponds to a different link length. In this case we obtained graphs with a clustering coefficient higher than that of the initial two-dimensional grid, in addition we significantly decrease the average path length and consequently the diameter of the graph.

**Table 1:** Optimistic condition. Mean values of the clustering coefficient, diameter and average path length of the initial graph and the final graphs with different link lengths under the optimistic rewiring rule.

Optimistic condition			
Link length	ACC	Diameter	APL
*Initial graph*	0	98	33.3333
*D*	0.1005	7	4.4488
*D*/2	0.1009	7	4.4226
*D*/4	0.1111	8.2	4.5745
*D*/8	0.1187	13.1	5.9231
*D*/16	0.1339	23.8	9.2273
*D*/32	0.2330	49	17.4089

We also observe that the more we limit the reach of the nodes to reconnect beyond their initial neighborhood, the more the graph tends to increase its clustering coefficient. On the other hand, we see that the more we limit the length of the link we increase the diameter and the average path length of the final graph.

In the Table 2 we observe the second set of experiments, using the Conservative condition. As we saw in previous results, as the link length goes shorter, the cluster coefficient increases and the diameter and average path length decrease. However, we also observed differences from the previous condition: the measured characteristics are bigger than those of the corresponding Optimistic condition. Unlike previous experiments, the clustering coefficient does not change significantly with different link lengths, the diameter and average path length remain unchanged until the link decreases beyond *D*/16.

**Table 2:** Conservative condition. Mean values of the clustering coefficient, diameter and average path length of the initial graph and the final graphs with different link lengths under the conservative rewiring rule.

Conservative condition			
Link length	ACC	Diameter	APL
*Initial graph*	0	98	33.3333
*D*	0.2620	30.3	11.9666
*D*/2	0.2620	30.1	11.9333
*D*/4	0.2629	29.6	11.9085
*D*/8	0.2622	30.4	11.9852
*D*/16	0.2639	32	12.4470
*D*/32	0.2342	49	17.4185

**Fig 3:**
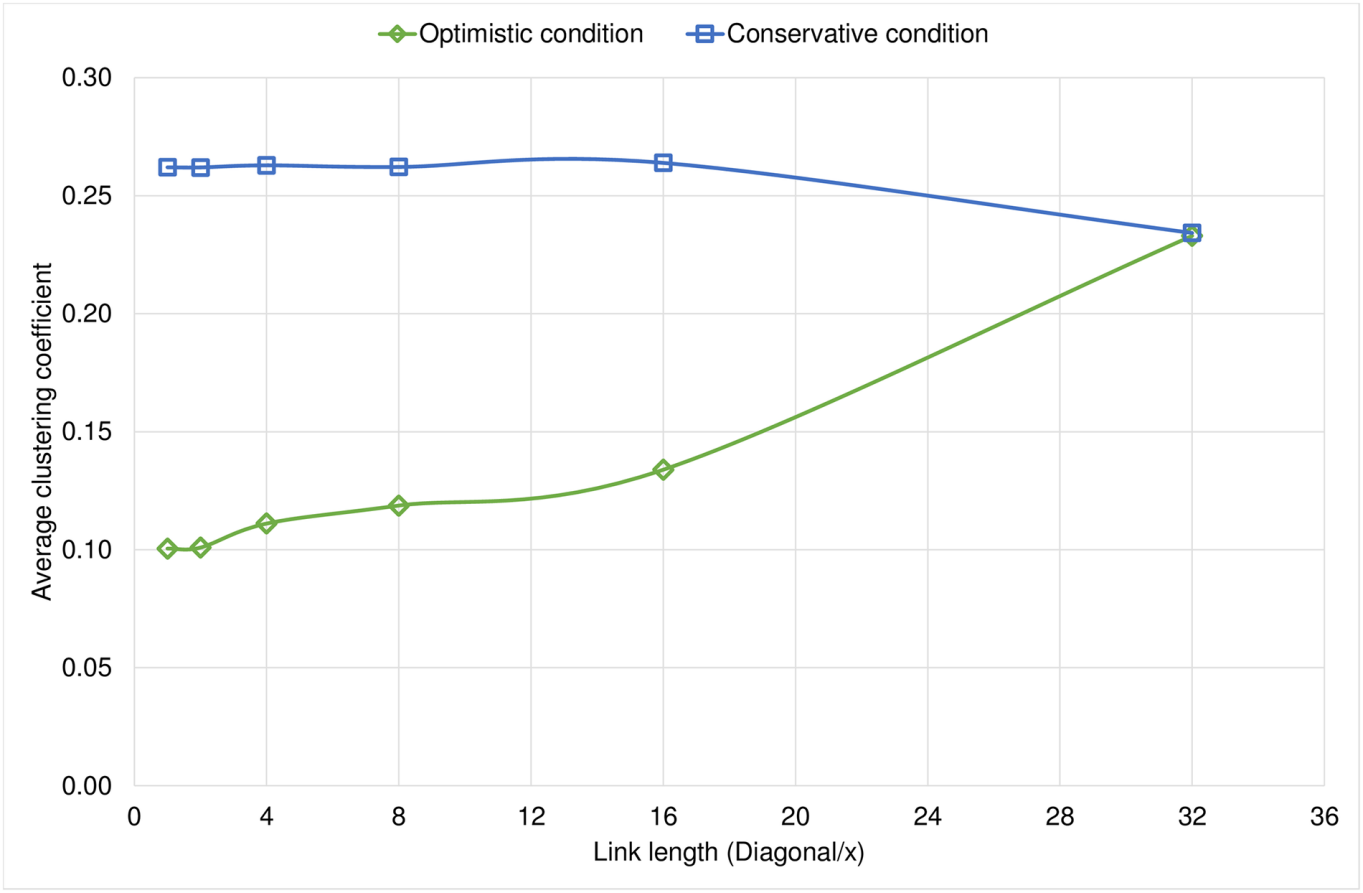
Comparison of the final average clustering coefficient for both rewiring conditions.

The rewiring condition allows us to create two different sets of graphs: in the first case, each node decides to rewire its links with less restrictions, so we see that the shortcuts formed achieve very short distances between nodes. Meanwhile, in the conservative experiments we see that with more restrictions, at the time of rewiring, we have larger distances between nodes, and it is possible that the nodes have more connections to their immediate communities, thus increasing the average clustering coefficient. In Fig 3 we observe that, under the Optimistic condition, a reduction on the scope of the node increases the average clustering coefficient.

**Fig 4:**
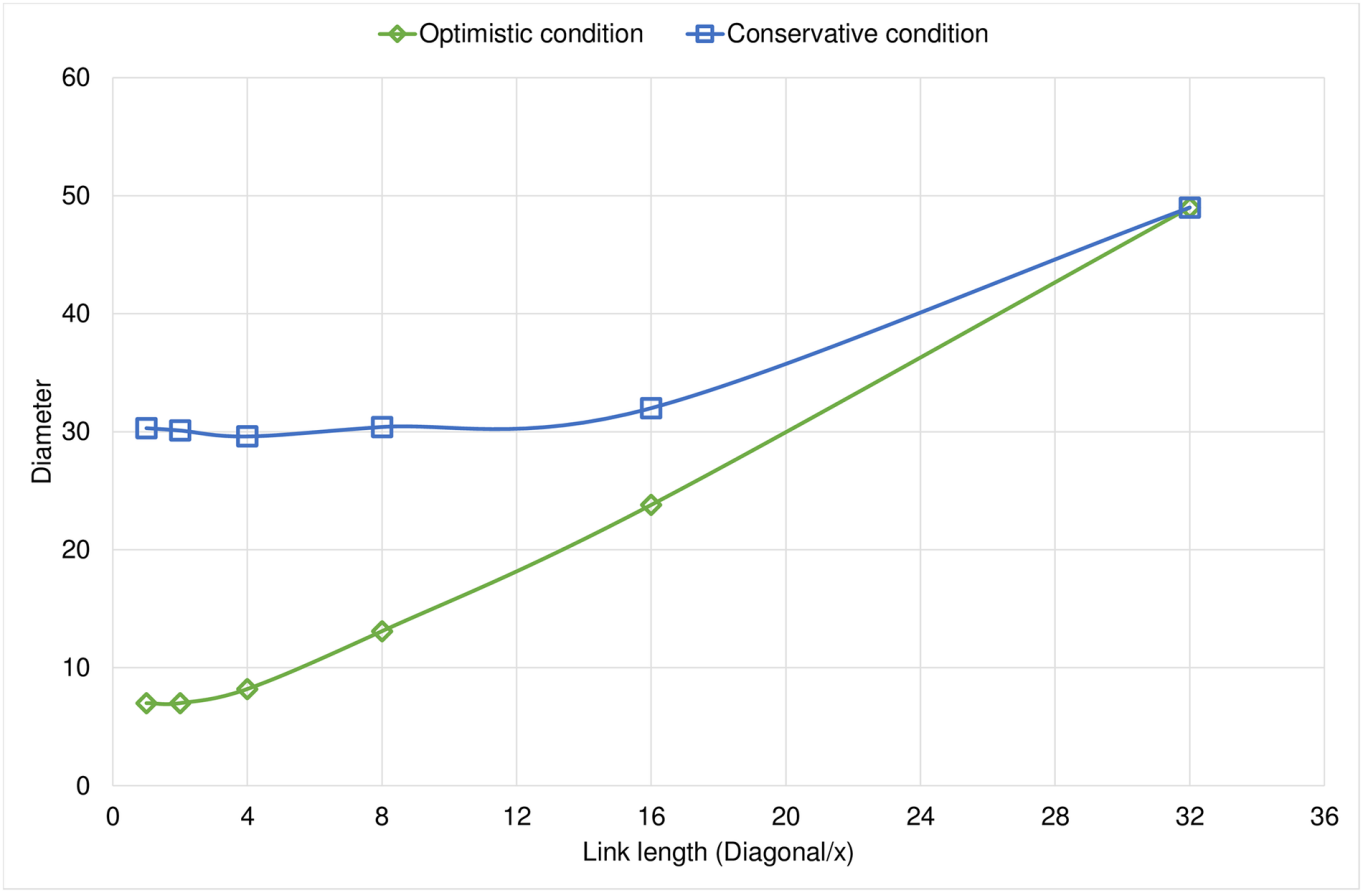
Comparison of the final diameter for both rewiring conditions.

**Fig 5:**
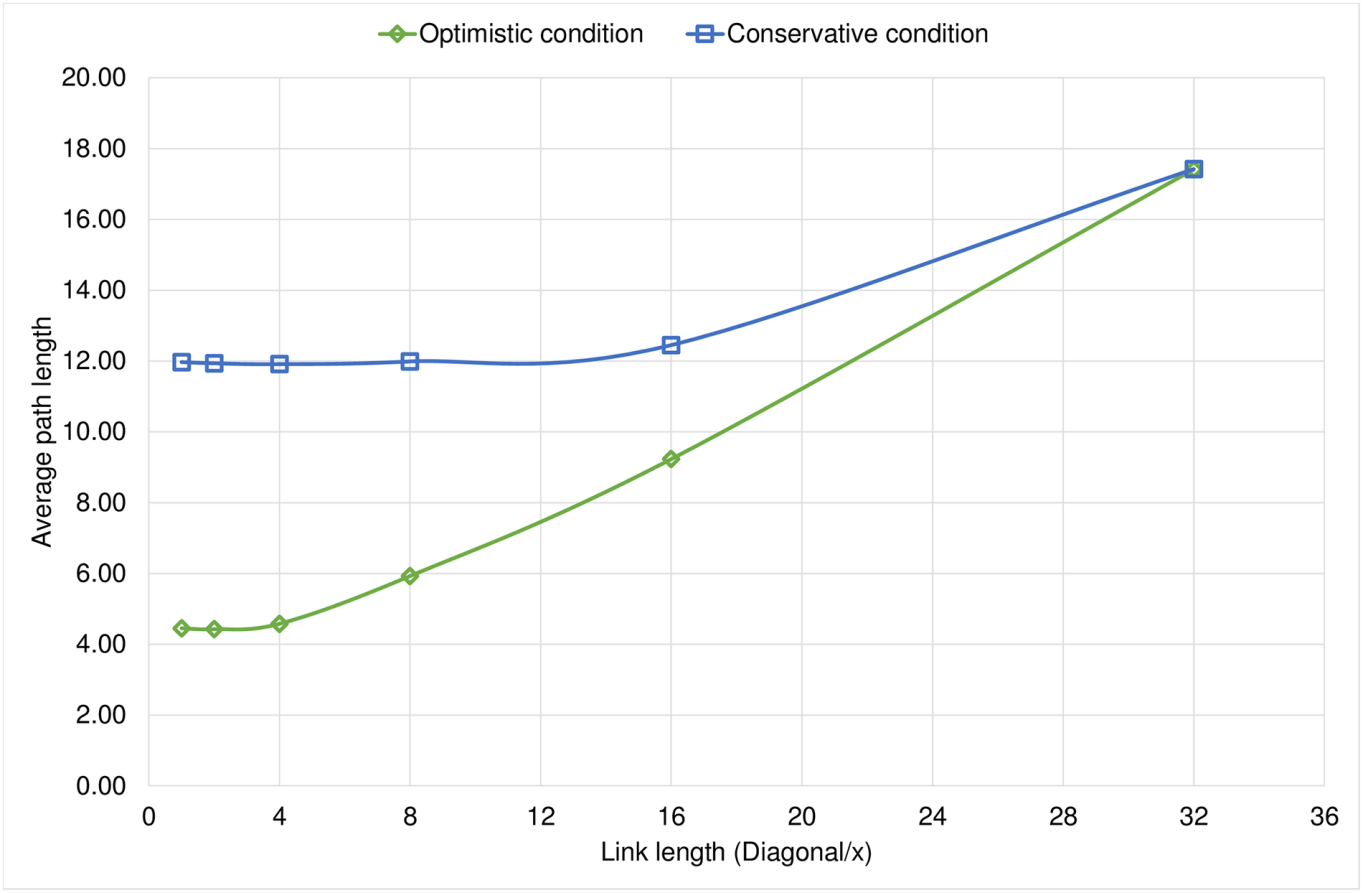
Comparison of the final average path length for both rewiring conditions.

An important result is that when the link length of a node is rather limited, we obtained graphs with similar characteristics in both families of experiments. This is because the nodes can only reach nearby neighborhoods and in this case the two conditions work similarly as they only reach a very limited number of nodes. Under this circumstances any node has less opportunities to rewire their links since it is unlikely to find a very promising node compared to the neighbors it already has. We observe the same behavior in Figs 4 and 5 in which the diameter and the average path length respectively increase when we limit the link length because we limit the ability to establish long-range shortcuts.

**Fig 6:**
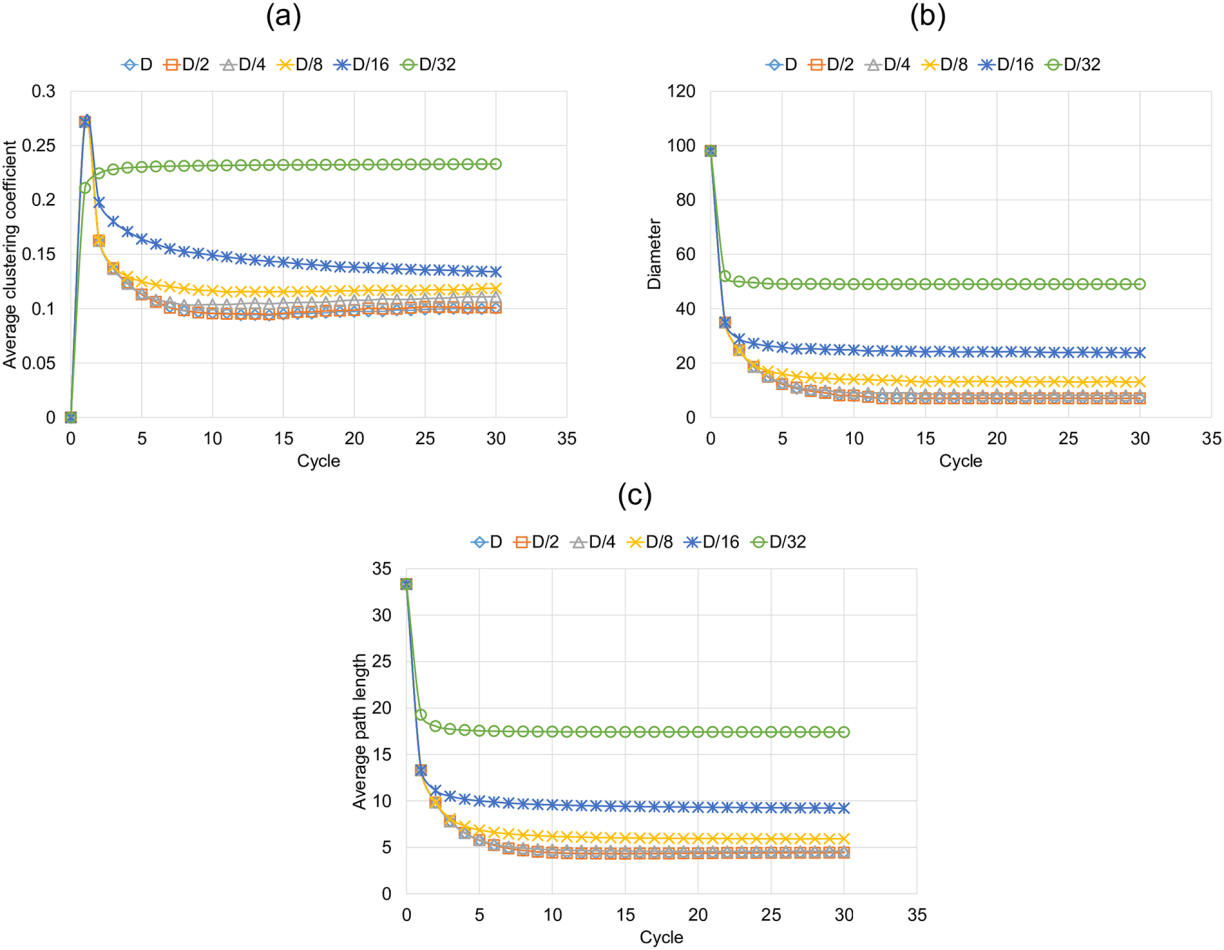
Characteristics of the graph for the optimistic rule and for each link length in each cycle of simulation. (a) Average clustering coefficient, (b) Diameter and (c) Average path length.

As mentioned in the previous section, the simulation advances through cycles in which, each node rewires some of its links so, the network and its characteristics evolve from a starting point to a final state. Fig 6 shows the shaping of the characteristics of the graph in each cycle, for the experiments corresponding to the Optimistic condition, with different link lengths. Fig 6a shows the average clustering coefficient (ACC), the range of this characteristic is [0, 1], with an initial value equal to 0. We observe that the final graph has its maximum average clustering coefficient, with link length equal to *D*/32. In most of cases we also observe that during the first cycle the ACC achieves a peak value that can be explained since all the nodes connect their temporal links resulting in a network with more links than the initial one, in which some initial neighborhoods are strengthened. Fig 6b and 6c show the diameter and the average path length, this measures behave in a similar way, in both cases we have the lowest values when the link length is longer, i.e. in *D*, *D*/2 and *D*/4. Also, it is important to mention that in the experiments that we carried out, it took less than fifteen cycles for these measures to stabilize.

**Fig 7:**
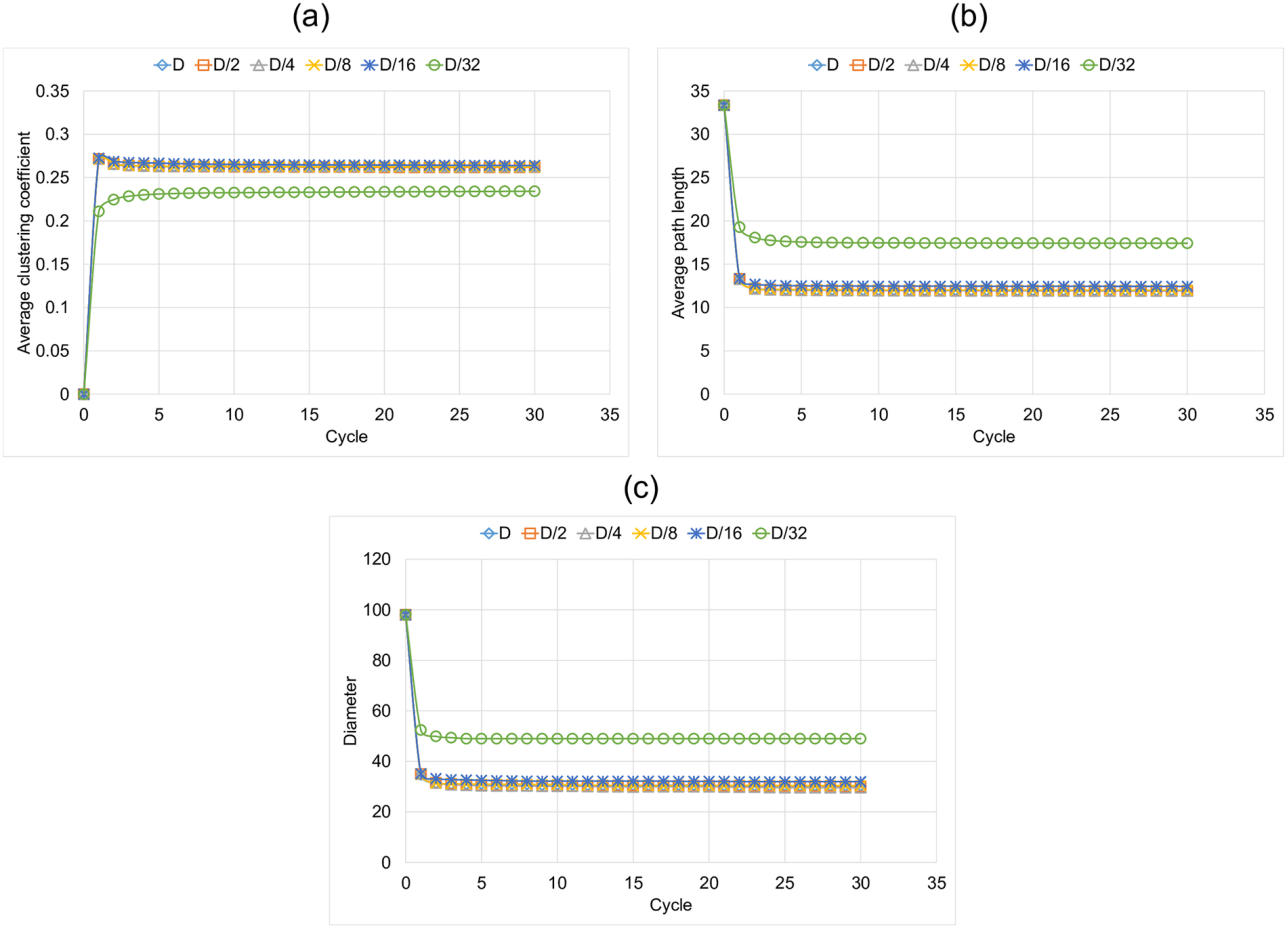
Characteristics of the graph for the Conservative condition and for each link length in each cycle of simulation. (a) Average clustering coefficient, (b) Diameter and (c) Average path length.

In turn, Fig 7 shows how the characteristics of the graphs change over time for the Conservative condition, under different link lengths. Unlike the previously mentioned experiments we see in Fig 7a that link length does not have a major impact on the final average clustering coefficient. Fig 7b and 7c show the evolution of diameter and average path length. In general, we achieve higher values than those corresponding to the Optimistic condition, but this time, we observe a similar behavior in almost all the experiments since the condition also adds restrictions that prevent the construction of shortcuts. It is interesting to see that, for both rewiring rules, the results corresponding to *D*/32 are almost identical. We considered that, in a scenario in which the temporal links are strongly constrained, a node chooses from a small set of nodes to rewire its links, so that it can only strengthen its immediate neighborhood for either of the two conditions.

**Fig 8:**
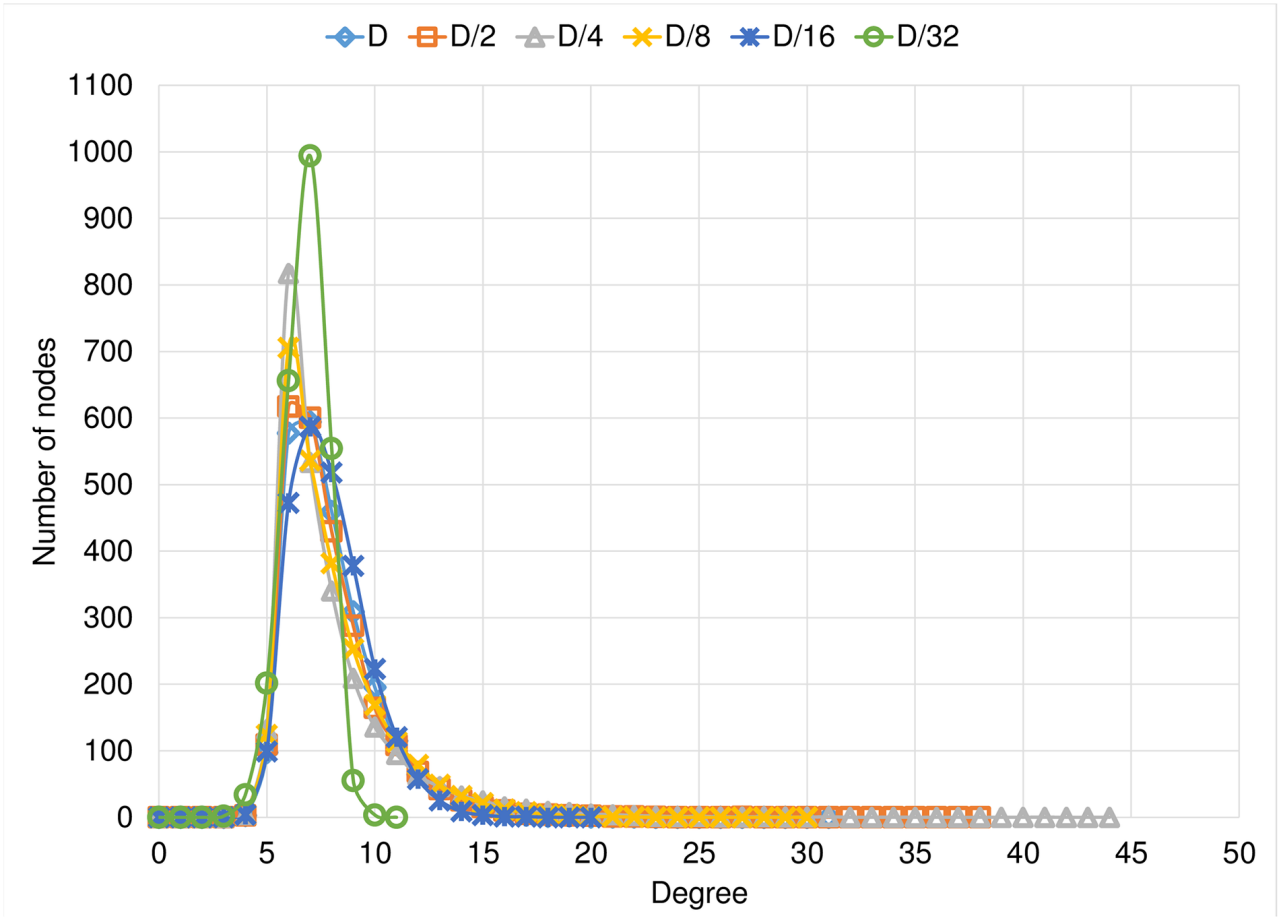
Degree distribution of the Optimistic condition experiments separated by the link length.

Another feature that provides with useful information about the emerging graphs is their final degree distribution. Fig 8 shows the mean degree distribution of the final graphs for the Optimistic condition experiments, for each link length tested. It is important to mention that there are no nodes with less than 4 links, because each node has 2 dynamic links and, at least, 2 fixed links. We observe that the highest-grade nodes have at most 44 neighbors in the Optimistic condition. As the link length goes larger, we have nodes with a larger number of neighbors, this is because these rich nodes or hubs can be reached by any node in the network, so more nodes become aware that these hubs are good shortcuts. Also, we can see that, shorter links prevent the arising of highly concentrated hubs. We show in the results the average histogram of the degree distributions. In the case of *D*/4, of the Optimistic condition, the *richest hub* appears very rarely. In these cases the richest node is in the center, and in the periphery of its area of reach a large number of small hubs are formed that connect it with the unreachable regions of the periphery of the grid. In another case, when we do not have a rich node, we only have one graph in which all the small hubs that are in the center are connected. In the cases of *D* and *D*/2, all nodes reach all the grid, so it is more complicated to choose a single important hub.

**Fig 9:**
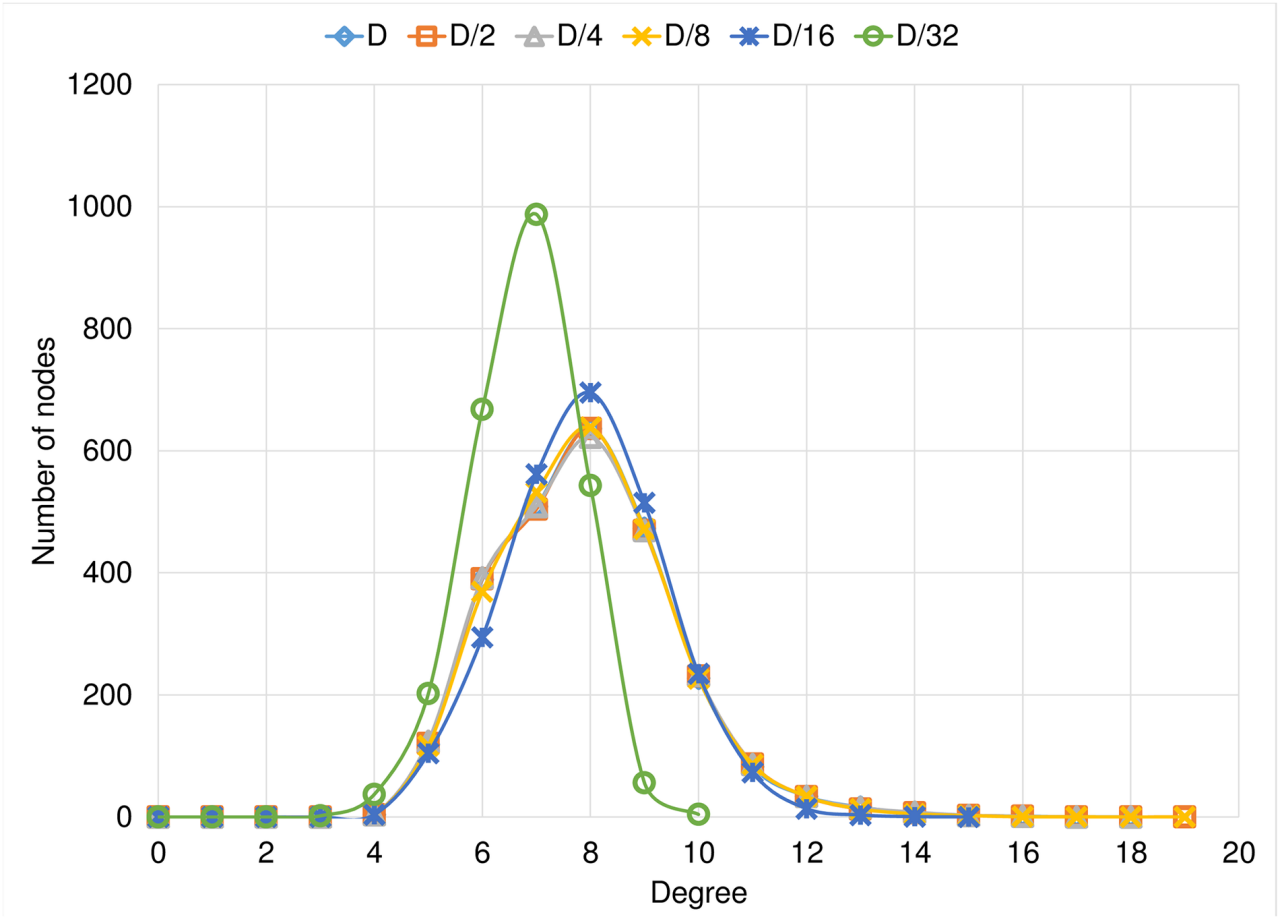
Degree distribution of the Conservative condition experiments separated by the link length.

Fig 9 shows the mean degree distribution of the final graphs for the Conservative condition experiments, for each link length tested. In this set of experiments, the highest-grade hubs are more limited, in all cases they have at most 19 neighbors, a very small number considering the graph order. We observed almost the same degree distribution for the experiments with link lengths equal to *D*, *D*/2, *D*/4, *D*/8 and *D*/16. For the case of more limited binding, there are even more limited hub nodes than in any other cases, most nodes in this case have between 4 and 10 neighbors.

**Fig 10:**
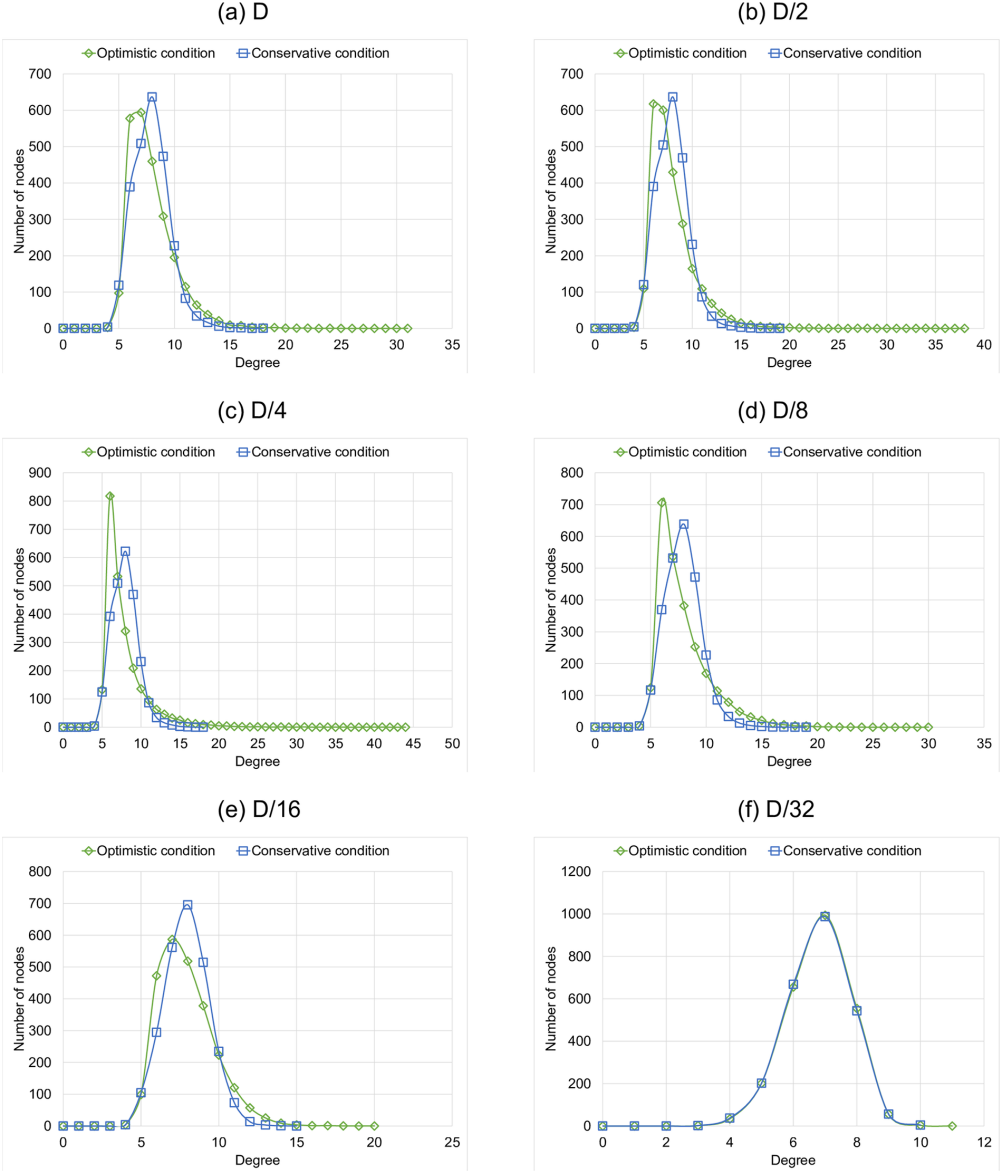
Degree distribution comparison between Optimistic condition vs Conservative condition.

Finally, in Fig 10 we compare the effect on the rewiring condition on the final degree distribution, for each link length tested. It is clear that the optimistic approach fosters the arising of high degree concentrators, a.k.a. *rich hubs*, provided that the link stretching allows the construction of long distance connections. Otherwise, i. e. under very short links, both strategies turn into a single one.

In addition to the comparison between experiments with the same rewiring condition, in Fig 10 we have a comparison of the degree distribution per link length. We observe that the final degree distribution depends very much on the rewiring rule. Being the Optimistic condition the one that produces *richer* hubs. With Fig 10F we reinforce the previously mentioned result in which we observe that, when we have a shorter link of length *D*/32, we obtained similar results for both conditions.

## Discussion

We proposed a rewiring model consisting of two main phases, the initial phase of partial information collection and the second phase of decision-making. This behavior is not strange to that of existing systems, for example in the work of Daniels et al. [38] we see that neuronal behavior consists of two phases similar to those that we propose. The first phase deployed to collect information from neighbor cells, and a second phase in which decisions are made.

One of the major choices in the distributed implementation of our model was the random designation of a node called *coordinator*, which announces the ending of each phase and the beginning of a new one on each cycle. This node is previously selected in the simulator configuration and can be any node in the network. A node always has locally updated information, but it is not always the case that it has valid system-wide information. Under these circumstances, it might be possible the overlapping between the packet exchange stage and the rewiring stage, preventing the successful completion of each phase and even inducing errors. For instance, if a node starts rewiring its links when tracers are still en route, many of the packets could be lost, and the corresponding source nodes would not be able to successfully update their view. Many of the packets could not find their recipients causing the analysis to be performed on fewer successful packet exchanges. Based on this analysis we decided to introduce a coordinator, which works like a global clock, announcing the ending of each stage and the beginning of the next. The coordinator is required to enforce a synchronized work which avoids the destruction of routes while they are still in use. For this purpose, the coordinator works pretty much like a Beta synchronizer [39]. It starts a broadcast procedure to announce the beginning of a new phase. After a certain time, it broadcasts a new message to announce the ending of the current phase. This procedure is followed by a feedback or convergecast procedure, where each node confirms the completion of its local task. Convergecast is also used to convey and gather information from the nodes to the coordinator. Right at the moment when the coordinator takes for granted that the phase is closed, and based on the information that it has just received, it decides whether to start a new phase or to stop the system permanently.

Another important decision for the proposed model was the introduction of two link types: fixed (undirected) and dynamic (directed). Our preliminary work showed that in a graph with a single type of edges (dynamic and undirected), we observed the arising of isolated components made up of the nodes of lesser use in general. That is, these nodes lost links as the simulation progressed. We decided to work with dynamic and directed links. When we talk about direction, we refer to the mechanism to avoid conflicts when a link is about to be rewired, that is to say, which of the two nodes connected by a given link is the only rightful owner of this resource. For any communication task, the edges have no direction. We obtained the diameter, average trajectory length and clustering coefficient considering an undirected graph.

From our perspective, the aforementioned decisions reflect an important lesson that we learned from the very beginning: it is necessary a minimal set of warranties to avoid disconnection during network reshaping.

The findings of this work show that we proposed a distributed rewiring model that creates networks with features similar to those found in complex networks that can be introduced in ICT systems such as P2P networks. In general, the resulting graphs have a higher average clustering coefficient, as well as a reduced diameter and average path length in comparison with the characteristics of the initial grid. In this work, we study the impact of two experimental settlements: 1) the rewiring rules each node follows (either conservative or optimistic) and 2) the reach of a node to rewire its links.

Regarding the results of the rewiring condition, we observe that the optimistic approach fosters the creation of shortcuts compared to the Conservative condition. These shortcuts bring about a reduction of diameter and average path length. However, the conservative rule gives us higher average clustering coefficients. As for grade distribution, we obtained higher grade hubs in the optimistic rule, than those arising in the Conservative condition. Regardless of the rewiring rule, a short link length seems to bound the reduction of the diameter and the average path length. On the other hand, we observe in this case a higher clustering coefficient than that achieved under longer link lengths.

Although each nodes acts looking for its individual benefit, we observed a global improvement on the network characteristics. This change is mainly because the nodes find the strategic points in the network that allow them to build shortcuts. These strategic nodes attract more connections as time goes by, and eventually turn into hubs. In most of the experiments, hubs are located near the center of the initial graph. For future work, it would be interesting to modify the initial settlement. In S1 Fig we observe four different cycles of an experiment with the experimentation tool using the Optimistic condition in a 10 × 10 grid.

We obtained high average clustering coefficients in two cases, when the link length is very short and when the nodes are under the Conservative condition. In the first case, this happens because each node can choose new neighbors within a very small area, increasing the clustering coefficient of the nodes lying at the range of the node. In the second case, in Conservative condition, if a node does not find a candidate that offers better chances, it will not move its links. Under these conditions, a node will create fewer links out of its known neighborhood than the Optimistic condition. That is because at the beginning of the simulation the probabilities of the nodes to be chosen are similar in both conditions but eventually the probability of a node holding its neighbors increases and the probability of creating long-range links decreases. Avoiding the formation of long-range links increases clustering coefficients, and consequently the resulting diameter is higher than those resulting from the Optimistic condition. In contrast, under the optimistic approach, we observe that the probability of rewiring is greater than that of maintaining a local neighborhood.

It is important to mention that, although we obtained degree distributions similar to that of Watts-Strogatz (WS), we observe certain differences. The proposed model is decentralized, although WS could also accept a decentralized version. One important difference is that the model WS does not consider distance. However, the most significant difference from their work is the probability of rewiring, which in WS is uniform. That is, any node can end up at the other end of a link, while our experiment indicates that the probability of being requested depends on the way in which its peers perceive the utility of a node. In this sense, our model has more coincidences with a preferential attachment.

We started this project searching for new techniques to study ICT networks. We think that our model does not only help to understand the structure of telecommunications networks, but it also could be applied to develop new rewiring strategies, as they are required in P2P systems, for instance. For further work, we consider the possibility of investigating different or complementary rewiring rules. For example in a telecommunications system, if a node becomes a hub it might turn into a potential bottleneck. What would be the emerging structure if a node limits the number of connections that can support? Is there a rewiring rule that may turn an arbitrary network into a navigable structure?

We know that the study of complex networks has become a multidisciplinary tool that is widely used in different areas of knowledge. It is fascinating to observe that many of our conclusions could be understood from an economic perspective, for instance. We hope that our findings could be used by this growing community of scientists and engineers.

## Supporting information

S1 Fig Example of real graph in the experimentation tool. Example of a resulting graph after some rewiring cycles in a 10 × 10 grid with Optimistic condition.
(TIF)
