# Supplementary figures and images for: Distributed rewiring model for complex networking: The effect of local rewiring rules on final structural properties
Source: PLoS One. 2017 Nov 6;12(11):e0187538. doi: 10.1371/journal.pone.0187538 (PMC5673200; doi:10.1371/journal.pone.0187538)

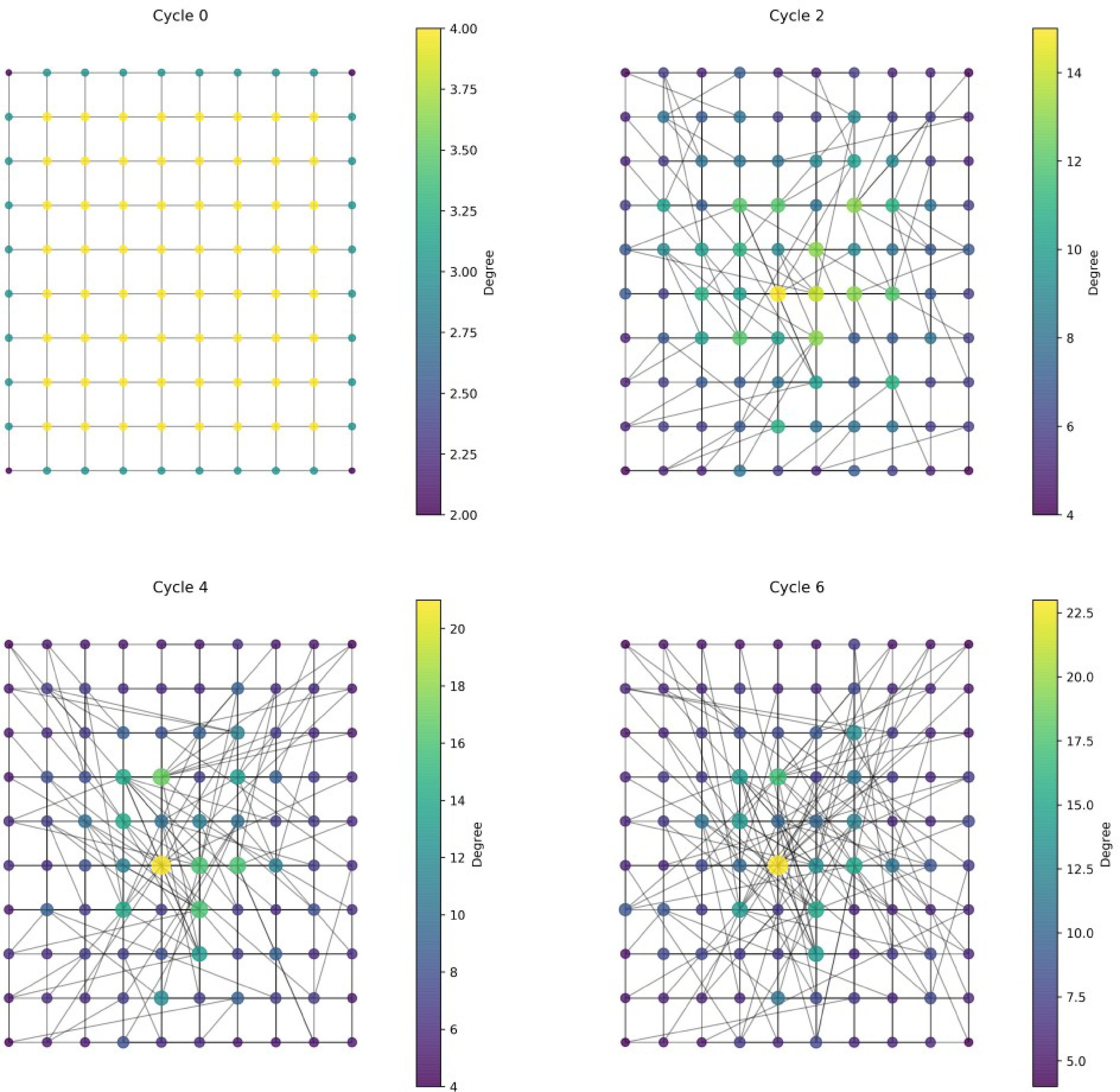

Supplement: S1 Fig — Example of a resulting graph after some rewiring cycles in a 10 × 10 grid with Optimistic condition. (TIF) [file pone.0187538.s001.tif]
